# GeoGenIE: a deep learning approach to predict geographic provenance of biodiversity samples from genomic SNPs

**DOI:** 10.1093/bioadv/vbaf250

**Published:** 2025-10-09

**Authors:** Bradley T Martin, Zachery D Zbinden, Michael E Douglas, Marlis R Douglas, Tyler K Chafin

**Affiliations:** Department of Biological Sciences, Seton Hall University, South Orange, NJ 07079, United States; Department of Biological Sciences, University of Arkansas, Fayetteville, AR 72701, United States; Department of Biological Sciences, University of Arkansas, Fayetteville, AR 72701, United States; Department of Biological Sciences, University of Arkansas, Fayetteville, AR 72701, United States; Department of Biological Sciences, University of Arkansas, Fayetteville, AR 72701, United States; Biomathematics and Statistics Scotland, Edinburgh EH9 3FD, United Kingdom; Tree of Life Programme, Wellcome Sanger Institute, Hinxton CB10 1SA, United Kingdom

## Abstract

**Motivation:**

Determining geographic origin of samples is a common objective in wildlife management, forensics, and conservation. Current methods often assume evolutionary models or require extensive reference datasets, which are costly and difficult to develop, that perform poorly with uneven or biased sampling. Supervised deep learning offers a promising alternative by learning complex patterns without prior model specifications. Combined with novel geo-genetic data augmentation and preprocessing techniques, it can reduce reference panel demands and improve performance across diverse sampling schemes, broadening accurate provenance determination to more study systems.

**Results:**

We present GeoGenIE, an open-source software package powered by PyTorch for geographic provenance prediction from genomic data. GeoGenIE implements a multilayer perceptron architecture within an automated hyperparameter tuning framework, incorporating preprocessing, geo-genetic outlier detection, and data augmentation to improve accuracy in sparsely sampled regions. Benchmarking against a comparable approach with White-tailed deer (*Odocoileus virginianus*) double digest restriction-site associated DNA sequencing data, GeoGenIE achieved substantially improved geolocation accuracy with less spatial bias using a smaller SNP panel. Gains were most evident in undersampled regions, underscoring effectiveness under challenging conditions. Its parallelized execution also produced fast runtimes, promoting its application to large datasets.

**Availability and implementation:**

Open-source at https://github.com/btmartin721/geogenie and https://pypi.org/project/GeoGenIE/.

## 1 Introduction

Genetic tools have been widely adopted for provenance determination (or natal location) of plant and animal tissues, with applications particularly prevalent in wildlife forensics (Ogden and Linacre 2015) and conservation (Breed *et al.* 2019). Dimension reduction techniques such as principal components analysis (PCA) have long been the method of choice for leveraging genetic spatial structure to infer provenance (e.g. Menozzi *et al.* 1978). This technique involves using linear transformations to project the data to a more easily visualized “embedded” space, wherein the provenance of unknown samples can be inferred based on their proximity to samples of known origin. However, distortions caused by spatial autocorrelation (Novembre and Stephens 2008) and other statistical artifacts (e.g. Podani and Miklos 2002) can drastically reduce the accuracy of inference, particularly when compounded by unmodeled biological processes, such as linkage disequilibrium (Baran *et al.* 2013).

Model-based methods, such as the spatial ancestry analysis (SPA) approach introduced by Yang *et al.* (2012), instead use theoretical assumptions of how allele frequencies are expected to behave in space and attempt to capture these mechanics within a probabilistic model. This class of methods has been widely adopted in spatial population genetics, with several different implementations (e.g. Bradburd *et al.* 2016, Guillot *et al.* 2016). Most assume a model describing how allele frequencies change spatially, such as the logistic function of Yang *et al.* (2012) or distance-based relatedness decay (e.g. Wasser *et al.* 2004).

While model-based inference has been the dominant paradigm in population genomics, the rapid proliferation of genome-scale datasets has fueled a transition toward an approach that is data-driven, rather than model-driven, especially via machine learning algorithms (Schrider and Kern 2018, Korfmann *et al.* 2023, Huang *et al.* 2024). LOCATOR, developed by Battey *et al.* (2020), introduced the first use (to our knowledge) of a machine learning approach for geolocation using high-dimensional genetic data. LOCATOR uses deep neural networks, which may be viewed as a class of generalized regressors that “learn” the underlying functional form of the data rather than relying upon a previously defined model (Warner and Misra 1996).

Departing from prior model specification offers a promising route to mitigate issues associated with violated assumptions, although it requires a large amount of balanced training data to implement effectively. Accordingly, many population genomic applications have relied upon simulations to supervise deep learning models (Borowiec *et al.* 2022). However, doing so can inadvertently reinforce the limitations of model-based inference by encoding model assumptions as an inductive bias of the learned network. This sort of “leakage” of model assumptions can lead to poor performance when the trained network is applied to empirical datasets.

Deep learning applications in population genomics have two complementary problems: (i) a need to generalize predictive performance beyond underlying biological model assumptions and (ii) a need for more data to train a network with reasonable performance. As used by LOCATOR (Battey *et al.* 2020), we propose a supervised deep learning approach, which seeks patterns directly from empirical data but leverages several automated extensions to improve performance: outlier detection, hyperparameter optimization, and novel extensions of existing data augmentation techniques (e.g. Chawla *et al.* 2002) for geo-genetic datasets.

We here present GeoGenIE, a novel software for geolocation prediction from genome-scale SNP datasets, implementing robust automated hyperparameter optimization, integrated geo-genetic outlier detection, and several approaches for mitigating uneven geographic sampling density. GeoGenIE offers substantial improvement over current state-of-the-art methods for geolocation by mitigating data-oriented biases frequently encountered in population genetic datasets (e.g. Chafin *et al.* 2021). In doing so, its utility encompasses a broader spectrum of study systems, especially those with sparse or spatially biased sampling, as well as those involving species with complex biogeographic histories or heavily impacted by human intervention (e.g. through translocation—human-mediated relocation of wildlife). Moreover, automated hyperparameter optimization maximizes the model’s predictive accuracy and simplifies use. As such, GeoGenIE reduces barriers to deployment in various wildlife management applications that may benefit from geographic provenance determination, from detecting the origin of diseased animals (Douglas *et al.* 2019) to tracking illegal translocations or poaching (Ogden *et al.* 2009).

## 2 Methods

### 2.1 Program description and interface

**Figure 1.**
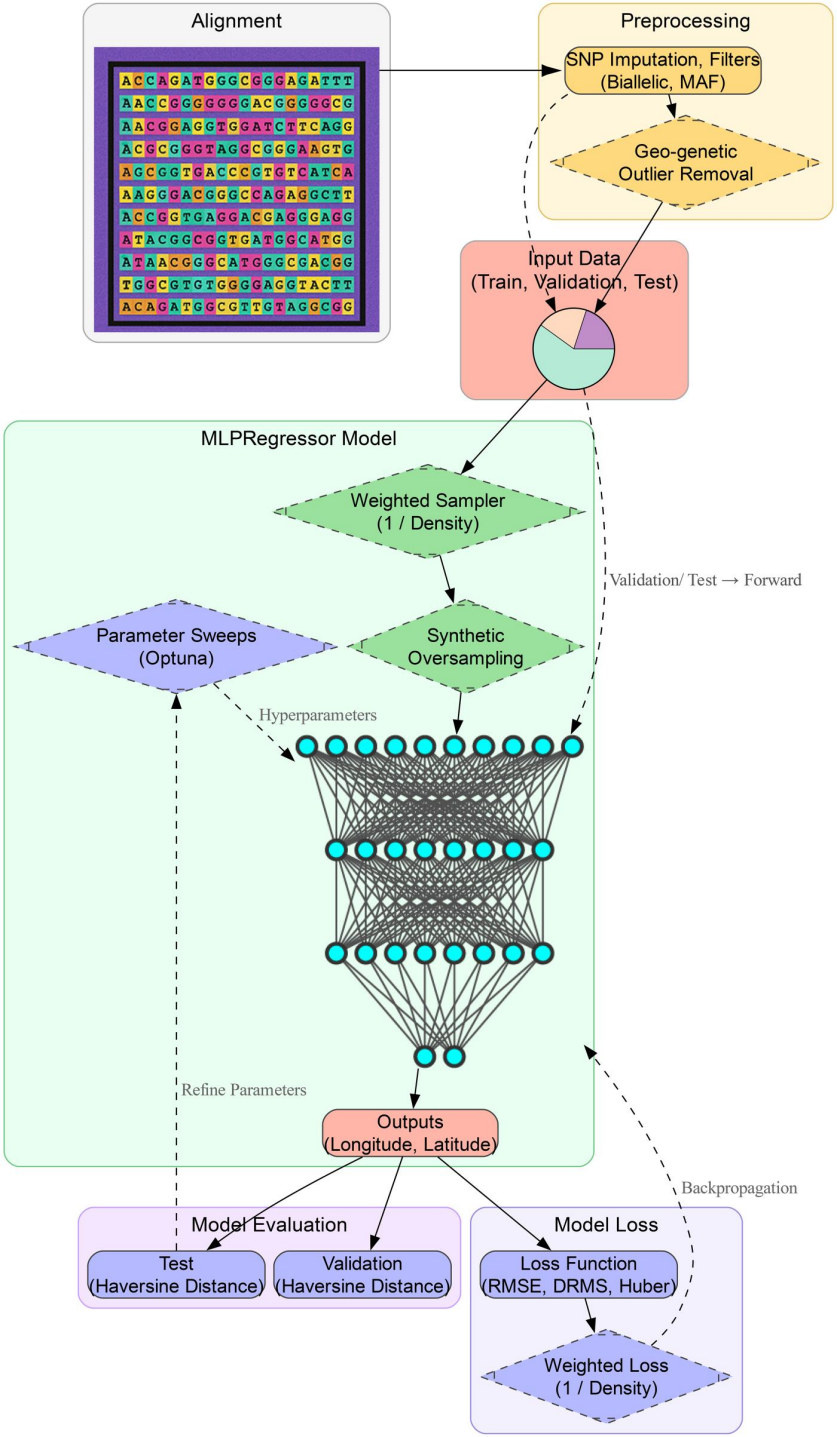
GeoGenIE workflow (diamond nodes = optional steps). The inputs get partitioned into training, validation, and test sets (pie chart), proceeded by outlier removal (training dataset). To address spatial bias, training samples optionally undergo an inverse density-based weighted resampling routine [1/(samples/km^2^)] and/or data augmentation (synthetic oversampling). The multilayer perceptron (MLP) neural network regressor illustrates GeoGenIE’s model architecture, which outputs geospatial coordinates (longitude and latitude). Loss is computed via root mean squared error (RMSE), distance root mean square (DRMS), or Huber, with optional weighting by inverse sampling density. Evaluation calculates pointwise Haversine (great circle) distance between predictions and ground truths.

To aid in the discovery of geographic “point of origin” for unknown genomic samples (i.e. provenance or natal location), we developed an open-source deep learning package, GeoGenIE, covering the full analytical workflow from missing data imputation to model inference and visualization (Fig. 1). User interaction is via a dedicated command-line interface (CLI), with runtime behavior and output plotting esthetics defined via a YAML configuration file or command-line arguments. The general workflow is as follows, with mathematical derivations for novel methodologies described in Appendices 1–3, available as supplementary data at *Bioinformatics Advances* online:

- **Data loading and pre-processing**: Loading input genotype matrix, including optional subsetting or filtering by minor allele count. Genotype matrices are additionally processed to impute missing states, with an optional dimensionality reduction step via various embedding strategies (e.g. multidimensional scaling).
- **Data augmentation and anomaly detection**: GeoGenIE implements several mitigation strategies to address spatial sample imbalance, including data augmentation and weighting methods and a geo-genetic outlier detection algorithm (Chang and Schmid 2023) based on isolation-by-distance expectations (for more detail, see Section 2.2 and Appendix 1, available as supplementary data at *Bioinformatics Advances* online).
- **Model training**: GeoGenIE uses a multilayer perceptron (MLP) architecture (Appendix 2, available as supplementary data at *Bioinformatics Advances* online), building upon that used by Battey *et al.* (2020), with data divided into test, train, and validation sets. Optionally, users can enable model parameter optimization using the Optuna framework (Akiba *et al.* 2019) to identify parameter combinations that improve predictive efficacy.
- **Prediction**: The trained model is then used to infer the geographic provenance of unknown samples, with optional bootstrapping to estimate geolocation confidence intervals.
- **Plotting and visualization**: GeoGenIE automatically outputs several visualizations of model diagnostics, ready-to-share plots of per-sample predictions (both bootstrapped and aggregated), and a variety of summary statistics.

To simplify implementation, GeoGenIE accepts the canonical Variant Call Format (VCF; Danecek *et al.* 2011) as its primary input, alongside a text file containing geographical coordinates (latitude/longitude) of known samples. Model training supports both CPU and GPU parallelization via PyTorch (Paszke *et al.* 2019), with other computationally expensive steps (e.g. parameter optimization, bootstrapping) utilizing CPU parallelization. The final trained model can be saved and re-loaded for subsequent inference-only runs without retraining on the full dataset.

### 2.2 Data preprocessing and augmentation

GeoGenIE incorporates several data preprocessing steps prior to model training, including feature and target encoding as well as normalization and augmentation. Several mitigative strategies are implemented to address the problem of spatial sampling imbalance frequently encountered in population genetic studies. First, sample weights are computed as the inverse geographic sampling density [i.e. 1/(samples/km^2^)], smoothed using kernel density estimation (KDE). These weights can optionally be used as a part of a weighted loss function (Appendix 3, available as supplementary data at *Bioinformatics Advances* online), penalizing poor predictions from regions of low sampling density, thus encouraging the model to focus learning on these areas. Second, we have implemented a novel data augmentation strategy inspired by the SMOTE algorithm (Chawla *et al.* 2002), tailored for spatial and genetic data (Appendix 1, available as supplementary data at *Bioinformatics Advances* online). First, samples are spatially clustered using *k*-means, with the optimal number of clusters (*k*) selected via mean silhouette width. To correct for sampling bias, we then apply a spatially constrained, cluster-aware nearest-neighbor interpolation algorithm that balances cluster densities. For each sample, synthetic genotypes are generated by identifying geographically proximate neighbors and hybridizing their genotypes using a Mendelian-inspired probabilistic model. This biologically grounded approach increases representation of sparse geographic clusters and improves robustness in downstream predictive modeling. As a final mitigative strategy, GeoGenIE uses a stratified random sampling approach to balance the representation of regions of high and low sampling density in the test-train-validation split. Here, cluster densities (as above) may be used to inform subsetting, thereby preventing the case that high-density areas dominate the model’s learned representation (He and Garcia 2009).

Another problem affecting many species for which GeoGenIE might be practically applied is the potential for past management actions such as translocation to have created “artificial” spatio-genetic patterns (Chafin *et al.* 2021), which can then leak into the learned model. To mitigate this, we have adapted a version of the GGOutlieR algorithm (Chang and Schmid 2023) as an automated optional step for anomaly detection. GGOutlieR operates by identifying genetic or geographical outliers deviating from the expected isolation-by-distance pattern (e.g. Slatkin 1987)—for example, those more (or less) geographically distant than expected, given their global genetic similarity in the sample set. GeoGenIE then prunes flagged outliers from the training dataset to eliminate their influence on the model.

### 2.3 Model architecture and training

GeoGenIE implements a multi-layer perceptron (MLP) architecture adapted from Battey *et al.* (2020) (Appendix 2, available as supplementary data at *Bioinformatics Advances* online), with some important differences. As in Battey *et al.* (2020), the network starts with a batch normalization layer and a user-specified number of hidden (i.e. internal) layers that facilitate learning of complex, non-linear patterns. Both models also use an Adam optimizer to handle backpropagation. However, in addition to the data augmentation techniques, we implemented three major MLP architecture additions over LOCATOR. (i) Dynamic scaling of hidden layer widths prevents overparameterization with few input features. If the initial layer width exceeds the product of the alignment dimensions (i.e. rows × columns), we recursively decrease its width by 20% and, once in compliance, use the final width uniformly across all hidden layers. (ii) The output from each hidden layer uses an Exponential Linear Unit (ELU) activation function. (iii) GeoGenIE supports three custom options for model loss functions: Root mean squared error (RMSE), Huber, and distance root mean square (DRMS), each of which can optionally be weighted by inverse sampling density to encourage model training to focus on sparsely sampled regions. (iv) Importantly, most model hyperparameters, such as the number of hidden layers and the learning rate, can be automatically chosen using a Bayesian optimization (tree-structured Parzen estimator) routine in Optuna (Akiba *et al.* 2019). This optimization procedure represents a crucial feature, as it simplifies user choices, thus requiring less deep learning domain knowledge to deploy, while also maintaining efficiency over brute-force searches that permute all possible combinations.

Other departures from LOCATOR include a scheduler to gradually decrease the learning rate during training and an early stopping mechanism that terminates training once validation error plateaus for a specified patience period (Appendix 3, available as supplementary data at *Bioinformatics Advances* online). This configuration accelerates convergence and reduces overfitting, which occurs when the model over-learns patterns specific to the training data, making it less able to generalize effectively to “unseen” data. Finally, GeoGenIE includes an additional validation dataset only assessed after completion of the training process to prevent data leakage and implements an optional gradient clipping strategy, which can reduce the “exploding gradient” problem described by Bengio *et al.* (1994).

## 3 Results

### 3.1 Case study: White-tailed deer (*Odocoileus virginanus*)

To demonstrate the utility of GeoGenIE, we used a publicly available dataset (PRJNA690954) representing reduced-representation double digest restriction-site associated DNA (ddRAD) sequencing data for *N = *1149 samples of White-tailed deer (*Odocoileus virginanus*) from throughout Arkansas, USA. The White-tailed deer study system represents a highly managed species due to its status as a commercially important game species and because they serve as a host to chronic wasting disease (CWD), a deadly and transmissible prion-based neurodegenerative disease. They are also a valuable case study for development of geolocation methodology, because they demonstrate several artifacts hindering straightforward analysis: substantially skewed distribution of sampling effort with heavy focus toward CWD-affected zones, geo-genetic outliers reflecting human-mediated translocation, and localized inflations of genetic divergence reflecting dynamic historic demographies (Chafin *et al.* 2021). These criteria make the White-tailed deer dataset an ideal case study to test the mitigative strategies implemented in GeoGenIE.

For the analyses, 10% of the White-tailed deer samples were randomly held out as a prediction dataset (i.e. those with “unknown” origin; *N = *115). The training datasets for GeoGenIE and LOCATOR comprised 75% of the samples (*N = *644). The remaining samples were then split into test and validation datasets, each consisting of a 12.5% sample subset (*N = *108 and *N = *109, respectively), or pruned with the outlier detection algorithm (*N = *28). The outlier detection algorithm removes samples violating isolation-by-distance assumptions, for example those individuals originating from human-mediated translocation.

A motivating factor for developing GeoGenIE was to facilitate geographic prediction for wildlife management, where constrained budgets often necessitate a more targeted sequencing approach, such as GTseq (Campbell *et al.* 2015). GTseq presents a cost-effective alternative to larger-scale population genomic sequencing methods but presents analytical challenges because it includes far fewer SNPs. Thus, all SNPs in the panel must be informative. LOCATOR previously demonstrated a high degree of spatial bias in prediction accuracy on ddRAD data for White-tailed deer, reflecting underlying sampling bias, even with a much larger SNP panel (*N = *5000; Chafin *et al.* 2021). Here, we compare the performance of GeoGenIE and LOCATOR with *N = *436 SNPs, reflecting the GTseq assay developed by Douglas *et al.* (2024). These were chosen by estimating population-wise divergence and selecting the 500 most differentiated SNPs, with *N = *436 being retained following quality control procedures. Here, per-locus FST was computed from ancestry proportions derived with sNMF (sparse non-negative matrix factorization; Frichot *et al.* 2014). The resulting SNP panel was used for training, validation, and prediction with both LOCATOR and GeoGenIE.

### 3.2 Model settings and experimental design

Predictive and runtime performance were gauged by comparing GeoGenIE with LOCATOR (Battey *et al.* 2020) using the same holdout sample set. LOCATOR was executed with a batch size of 64 and otherwise default parameter settings. The results were compared against all combinations of the following GeoGenIE configurations: (i) Geo-genetic outlier detection enabled or disabled; (ii) equal or density-inverted sample weights, applied to a custom weighted loss function; and (iii) with or without SMOTE-based synthetic oversampling. All analyses were evaluated across 100 independent bootstrap pseudoreplicates.

GeoGenIE includes settings for fine-tuning performance and plot esthetics in addition to the three key mitigation strategies outlined above. We ran GeoGenIE on the White-tailed deer dataset using the following parameter settings: (i) maximum nearest neighbors = 20, considered in the outlier detection and sample weighting algorithms; (ii) number of spatial bins = 6, used in the synthetic oversampling algorithm, (iii) sample weight normalization enabled, (iv) the kernel density estimation option for calculating sample weights, and (v) a batch size of 64. These fixed options were only used when applicable (e.g. spatial binning was only applied with oversampling enabled). All other options remained default.

### 3.3 Performance comparison with other software

**Figure 2.**
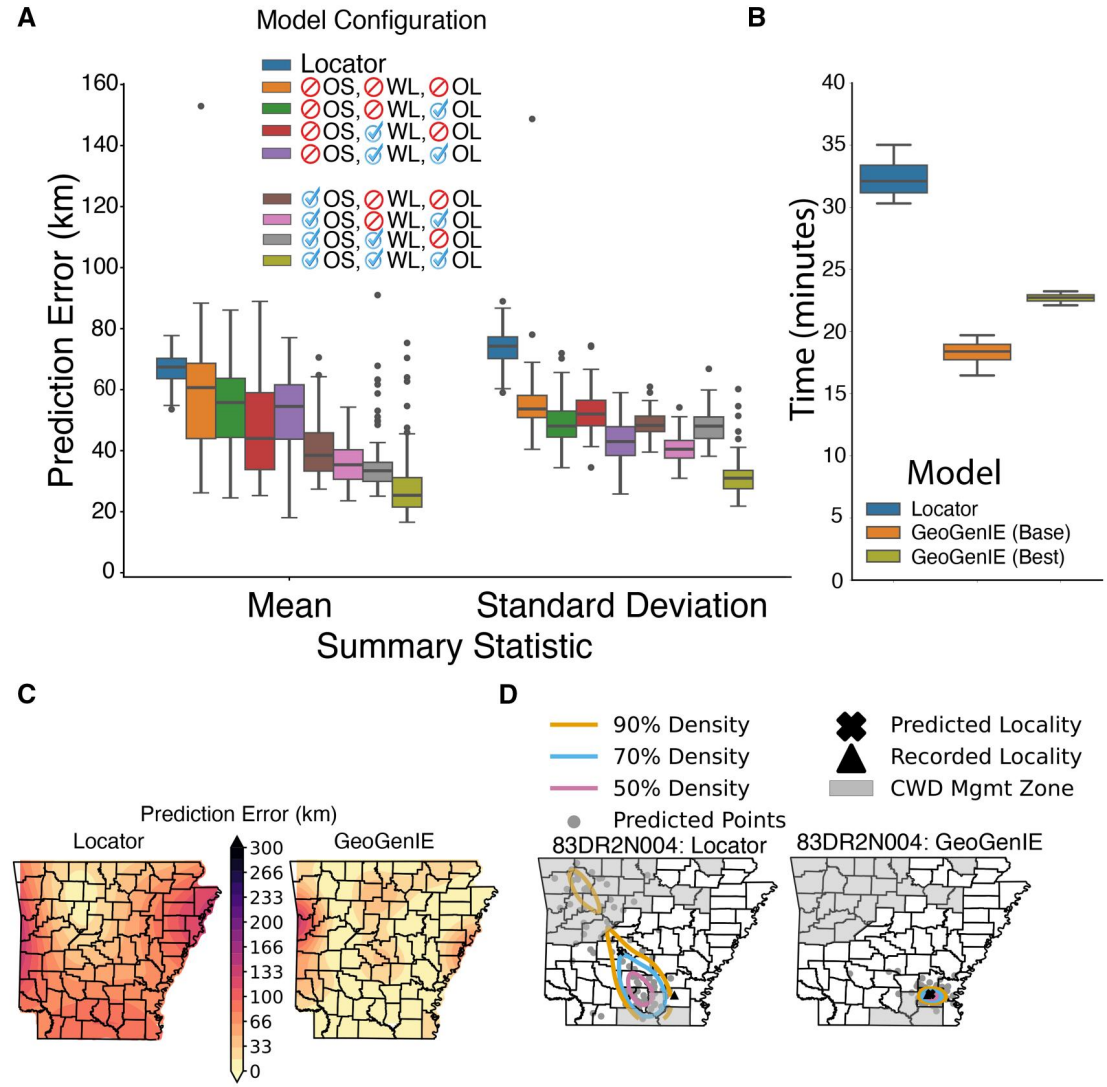
LOCATOR versus GeoGenIE model performance comparison among *N *= 100 bootstrap replicates with *N *= 436 SNPs and *N *= 108 held-out white-tailed deer samples. Prediction error represents Haversine distance in kilometers between predicted and recorded collection localities. (A) Box plots (via Seaborn v0.13.2; Waskom 2021) depict aggregated error for LOCATOR and all combinations of three GeoGenIE settings: oversampling = OS, weighted loss = WL, and outlier detection = OL. Blue check marks and red crossed circles indicate enabled and disabled settings. (B) Total execution time with *N *= 100 bootstrap replicates, measured across 10 full runs using the Hyperfine benchmarking software. (C) Spatial kriging interpolation of mean prediction error with the optimal GeoGenIE model (Panel A). (D) Prediction localities per bootstrap replicate (gray points) for a representative sample (83DR2N004), with their geographic centroid (✖ marker) and recorded collection locality (▲ marker). Contours enclose three levels of bootstrap prediction densities. Gray filled counties demarcate the chronic wasting disease (CWD) management zone (as defined in 2024).

Using the White-tailed deer dataset, we compared the geolocation performance of LOCATOR and GeoGenIE models, with prediction error measured as the Haversine (great circle) distance [i.e. a geospatial distance metric accounting for Earth’s curvature (Sinnott 1984)] between predicted and recorded tissue collection localities (Fig. 2A; Table S1, available as supplementary data at *Bioinformatics Advances* online). The mean prediction error for LOCATOR was 59.34 ± 16.88 km, while the GeoGenIE “base” model (with all mitigation strategies disabled) achieved a lower error of 51.29 ± 17.77 km. GeoGenIE’s best configuration, with all three mitigation strategies enabled, reduced the error to 26.37 ± 10.98 km, representing a 2.25-fold improvement over LOCATOR.

GeoGenIE’s mitigation strategies generally improved accuracy as more strategies were implemented (Fig. 2A). Oversampling produced the greatest prediction error reduction, with mean errors ranging from 26.37 ± 10.98 km to 38.36 ± 13.38 km. Configurations without oversampling exhibited higher errors, ranging from 44.84 ± 13.98 km to 51.29 ± 17.77 km. Weighted loss functions provided the second most significant improvement, reducing errors to 43.96 ± 16.67 km and 44.84 ± 13.98 km, depending on the outlier detection setting. When oversampling was enabled, errors improved further, reaching 26.37 ± 10.98 km and 34.43 ± 14.65 km.

Per-bootstrap runtimes were lower for LOCATOR, which completed individual pseudoreplicates (including training, evaluation, and prediction) in 39.5 ± 102.28 s. GeoGenIE’s fastest configuration, with sample weighting and outlier detection enabled, but no oversampling, completed iterations in 63.4 ± 34.1 s. Turning off weighted loss slightly increased runtime to 65.6 ± 27.7 s. However, adding SMOTE-based oversampling significantly extended training times, with runtimes ranging from 160.6 ± 52.4 s to 185.9 ± 60.7 s, depending on the settings. We additionally benchmarked total runtimes (e.g. across all bootstraps) using Hyperfine (Peter 2023) across 10 replicate runs, each with 100 bootstrap replicates (Fig. 2B). Despite longer per-bootstrap runtimes, GeoGenIE’s parallelization (here across eight CPUs) resulted in lower total execution times than LOCATOR. The base GeoGenIE model completed in 18.27 ± 0.97 min, while the best-performing GeoGenIE configuration (oversampling, weighted loss, and outlier detection enabled) required 22.7 ± 0.37 min. By comparison, LOCATOR took 32.26 ± 1.52 min. Optional hyperparameter tuning increases runtime across GeoGenIE configurations, here requiring 100 additional model training and evaluation steps, but has been observed to consistently result in better geolocation accuracy. Note that GeoGenIE supports much larger-scale parallelization than explored herein, as well as GPU model training, which will significantly impact total runtime.

GeoGenIE’s best-performing model outperformed LOCATOR in geographic provenance prediction across the sampling area (Fig. 2C), particularly in undersampled regions with complex translocation histories (Chafin *et al.* 2021; Fig. 2D). Of note, prior evaluations of LOCATOR on this dataset, using a larger number of SNPs (*N *= 5000 SNPs compared to *N *= 436 here) showed consistently poor performance results in undersampled regions (Chafin *et al.* 2021).

## 4 Conclusions

We here demonstrated that GeoGenIE achieves improved geolocation accuracy using the same reference sampling scheme as previous applications (i.e. Chafin *et al.* 2021), and with a notably smaller SNP panel (*N = *436 herein versus *N = *5000). Financial and human resources are the primary limiting factors in deploying molecular tools in conservation, necessitating a pragmatic approach that maximizes the sampling efficiency needed to guide management plans sufficiently (Bertola *et al.* 2024). The improved prediction performance of GeoGenIE promotes a substantially lower sampling effort with a comparable level of accuracy to previously available tools (Douglas *et al.* 2024), rendering it cheaper to deploy at scale and lowering the cost barrier to reference panel construction. Finally, we engineered GeoGenIE to be user-friendly, efficient, and fully automated wherever feasible to broaden its utility to the scientific community, simplify decision-making, and lower the required knowledge barrier. Together, these innovations position GeoGenIE as a scalable and accessible tool for advancing spatial genomic inference in both research and applied conservation contexts.

## Supplementary Material

vbaf250_Supplementary_Data

## Data Availability

GeoGenIE is hosted as a repository on GitHub (https://github.com/btmartin721/GeoGenIE) and can be installed via the Python Package Index (https://pypi.org/project/GeoGenIE). Raw sequences are accessioned in the National Center for Biotechnology Information Sequence Read Archive (NCBI; BioProject PRJNA690954) at (https://www.ncbi.nlm.nih.gov/bioproject/?term=PRJNA690954), and all assembled SNP alignments, study metadata, and results files are available in an Open Science Framework repository (https://doi.org/10.17605/OSF.IO/JSQZ9). The GeoGenIE code documentation can be found on Read the Docs (https://geogenie.readthedocs.io/latest/).
