# Supplementary material for: GeoGenIE: a deep learning approach to predict geographic provenance of biodiversity samples from genomic SNPs
Source: Bioinform Adv. 2025 Oct 9;5(1):vbaf250. doi: 10.1093/bioadv/vbaf250 (PMC12596584; doi:10.1093/bioadv/vbaf250)
Supplement: vbaf250_Supplementary_Data [file vbaf250_supplementary_data.zip › GeoGenIE_Supplementary_Appendices_v1.3.pdf]

Supplementary Appendices for GeoGenIE

Bradley T. Martin *et al.*

September 26, 2025

Supplementary Table S1: LOCATOR versus GeoGenIE model performance comparison among $N = 100$ bootstrap replicates with $N = 436$ SNPs and $N = 108$ held-out White-tailed deer (*Odocoileus virginianus*) samples. Prediction error represents Haversine distance in kilometers between predicted and recorded collection localities. Descriptive statistics of aggregated error is reported for LOCATOR and all combinations of three GeoGenIE settings: Oversampling, Weighted Loss, and Outlier Detection.

Configuration	Mean	StdDev	Minimum	25% Quantile	50% Quantile	75% Quantile	Maximum
Locator	59.34	16.88	25.47	40.63	65.55	72.36	88.95
No Oversampling, No Weighted Loss, No Outlier Detection	51.29	17.77	7.72	44.44	53.06	60.62	152.92
No Oversampling, No Weighted Loss, Outlier Detection	48.35	13.61	9.64	41.65	48.73	56.27	86.08
No Oversampling, Weighted Loss, No Outlier Detection	43.96	16.67	6.36	31.97	47.02	54.57	88.93
No Oversampling, Weighted Loss, Outlier Detection	44.84	13.98	5.94	37.65	45.78	54.52	77.05
Oversampling, No Weighted Loss, No Outlier Detection	38.36	13.38	8.11	28.94	40.61	48.46	70.55
Oversampling, No Weighted Loss, Outlier Detection	32.89	10.25	8.73	26.06	35.38	40.48	54.28
Oversampling, Weighted Loss, No Outlier Detection	34.43	14.65	6.84	21.11	34.45	46.28	91.00
Oversampling, Weighted Loss, Outlier Detection	26.37	10.98	7.54	19.19	26.04	31.62	75.31

Supplementary Appendix 1: Synthetic Genotype Interpolation via Nearest Neighbors

Authors: Bradley T. Martin *et al.*

To mitigate sampling bias and augment underrepresented spatial clusters in genotype datasets with GeoGenIE, we developed a synthetic genotype interpolation method inspired by the SMOTE (Synthetic Minority Over-sampling Technique) framework (**?**), tailored for population genomic data. This approach generates biologically realistic synthetic individuals by interpolating genotypes and geographic coordinates between spatially proximate samples.

Let

$$\mathbf{X} = \{\mathbf{x}_1, \mathbf{x}_2, \ldots, \mathbf{x}_n\} \in \mathbb{R}^{n \times m} \tag{1}$$

denote the genotype matrix, where each row $\mathbf{x}_i$ is a genotype vector of length m (e.g., encoded as $0, 1, 2$ for biallelic SNP loci), and let

$$\mathbf{Y} = \{\mathbf{y}_1, \mathbf{y}_2, \ldots, \mathbf{y}_n\} \in \mathbb{R}^{n \times d} \tag{2}$$

denote the corresponding matrix of (normalized) geographic coordinates for each sample.

We first cluster individuals based on their geographic coordinates using either k-means or kernel density estimation (KDE), yielding cluster labels

$$\mathcal{C} = \{c_1, c_2, \ldots, c_n\}, \quad c_i \in \{1, \ldots, K\}. \tag{3}$$

To address class imbalance, each cluster $\mathcal{C}_c$ is oversampled such that its size matches that of the largest cluster $\mathcal{C}_{\max}$. Specifically, for each individual i in cluster $\mathcal{C}_c$, we generate k_i synthetic samples, where

$$k_i = \left\lfloor \frac{|\mathcal{C}_{\max}|}{|\mathcal{C}_c|} \right\rfloor. \tag{4}$$

Genotype interpolation

For each synthetic sample, we select a neighboring individual $\mathbf{x}_j \in \mathcal{N}_i$ from within the same cluster or among geographically nearby neighbors. Genotypes are interpolated at each locus ℓ using a Mendelian-inspired inheritance model:

$$\tilde{g}^{(\ell)} \sim \text{Mendelian}\left(g_i^{(\ell)}, g_j^{(\ell)}\right), \tag{5}$$

where $g_i^{(\ell)}$ and $g_j^{(\ell)}$ are the diploid genotypes at locus ℓ for individuals i and j (coded $0, 1, 2$). The probabilities follow classic diploid Mendelian ratios, e.g.:

- If $g_i = 0$ and $g_j = 2$, then $\mathbb{P}(\tilde{g} = 1) = 1.0$;
- If $g_i = 1$ and $g_j = 1$, then $\mathbb{P}(\tilde{g} = 0) = 0.25$, $\mathbb{P}(\tilde{g} = 1) = 0.5$, $\mathbb{P}(\tilde{g} = 2) = 0.25$;
- If $g_i = g_j$, then $\tilde{g}^{(\ell)} = g_i^{(\ell)}$.

The synthetic geographic coordinates and sample weights are computed as arithmetic means of the parental values:

$$\tilde{\mathbf{y}} = \tfrac{1}{2}\left(\mathbf{y}_i + \mathbf{y}_j\right), \qquad \tilde{s} = \tfrac{1}{2}\left(s_i + s_j\right). \tag{6}$$

Each synthetic individual is represented as the triplet $(\tilde{\mathbf{x}}, \tilde{\mathbf{y}}, \tilde{s})$, and the augmented dataset is

$$\mathcal{D}' = \mathcal{D} \cup \{(\tilde{\mathbf{x}}, \tilde{\mathbf{y}}, \tilde{s})\}. \tag{7}$$

This procedure increases the representation of under-sampled spatial regions while preserving spatial–genetic structure and biological plausibility. It enables more robust population genomic inferences by reducing geographic sampling bias without introducing unrealistic genotype combinations.

Supplementary Appendix 2: Mathematical Description of the GeoGenIE Multi-Layer Perceptron

Authors: Bradley T. Martin *et al.*

Model Architecture Overview

The model is a Multi-Layer Perceptron (MLP) for regression that maps an input vector $\mathbf{x}$ to an output vector $\hat{\mathbf{y}}$. The architecture is defined by input size d_{in}, output size d_{out}, number of hidden layers L, hidden width h, and dropout probability p. The network includes (i) optional input **batch normalization**, (ii) an input (affine) layer, (iii) L hidden layers with ELU activations, (iv) dropout after the first $\lfloor L/2 \rfloor$ hidden layers, and (v) a linear output layer.

Mathematical Formulation

Let $\mathbf{x} \in \mathbb{R}^{d_{\text{in}}}$. The input may pass through batch normalization (BN):

$$\mathbf{a}^{(0)} = \begin{cases} \text{BN}(\mathbf{x}) & \text{if batch size} > 1, \\ \mathbf{x} & \text{otherwise.} \end{cases} \tag{8}$$

First hidden layer:

$$\mathbf{a}^{(1)} = \sigma_{\text{ELU}}\left(W^{(1)}\mathbf{a}^{(0)} + \mathbf{b}^{(1)}\right), \tag{9}$$

with ELU activation $\sigma_{\text{ELU}}(z) = \begin{cases} z, & z > 0 \\ \alpha(e^z - 1), & z \leq 0 \end{cases}$ (typically $\alpha = 1$). Subsequent hidden layers for $l = 2, \ldots, \lfloor L/2 \rfloor$:

$$\mathbf{a}^{(l)} = \sigma_{\text{ELU}}\left(W^{(l)}\mathbf{a}^{(l-1)} + \mathbf{b}^{(l)}\right). \tag{10}$$

Dropout is applied after layer $\lfloor L/2 \rfloor$ with probability p:

$$\mathbf{a}^{(\lfloor L/2 \rfloor, \text{drop})} = D\left(\mathbf{a}^{(\lfloor L/2 \rfloor)}, p\right). \tag{11}$$

The remaining hidden layers continue from $\mathbf{a}^{(\lfloor L/2 \rfloor, \text{drop})}$:

$$\mathbf{a}^{(l)} = \sigma_{\text{ELU}}\left(W^{(l)}\mathbf{a}^{(l-1)} + \mathbf{b}^{(l)}\right), \quad l = \lfloor L/2 \rfloor + 1, \ldots, L. \tag{12}$$

Output layer (no activation, regression):

$$\hat{\mathbf{y}} = W^{(\text{out})}\mathbf{a}^{(L)} + \mathbf{b}^{(\text{out})}. \tag{13}$$

Supplementary Appendix 3: Mathematical Formulation of Custom Training Components

Authors: Bradley T. Martin *et al.*

Custom Loss Functions

Weighted Distance Root Mean Square (DRMS) Loss

Given N samples with predicted coordinates $\hat{\mathbf{y}}_i = (\hat{\lambda}_i, \hat{\phi}_i)$ and true coordinates $\mathbf{y}_i = (\lambda_i, \phi_i)$ in degrees, and weights w_i, convert to radians and compute Haversine distances

$$a_i = \sin^2\left(\frac{\Delta\phi_i}{2}\right) + \cos(\hat{\phi}_i)\cos(\phi_i)\sin^2\left(\frac{\Delta\lambda_i}{2}\right), \qquad d_i = R \cdot 2 \cdot \operatorname{atan2}\big(\sqrt{a_i}, \sqrt{1-a_i}\big)\,. \tag{14}$$

The weighted DRMS is

$$\mathcal{L}_{\text{DRMS}} = \sqrt{\frac{1}{\sum_{i=1}^{N} w_i} \sum_{i=1}^{N} w_i d_i^2}, \tag{15}$$

which reduces to the unweighted DRMS when $w_i \equiv 1$.

Weighted Root Mean Square Error (RMSE) Loss

Per-sample Euclidean distance $d_i = \sqrt{\sum_j (\hat{y}_{ij} - y_{ij})^2}$ and batch mean $\bar{d} = \frac{1}{N}\sum_i d_i$. The (optionally) weighted loss is

$$\mathcal{L}_{\text{RMSE}} = \bar{d} \cdot \left(\frac{1}{N}\sum_{i=1}^{N} w_i\right), \tag{16}$$

with $\mathcal{L}_{\text{RMSE}} = \bar{d}$ if no weights are used.

Weighted Huber Loss with Target Smoothing

Targets are smoothed via ϵ toward the batch mean $\bar{\mathbf{y}}$:

$$\mathbf{y}_i' = (1-\epsilon)\mathbf{y}_i + \epsilon\,\bar{\mathbf{y}}, \qquad \bar{\mathbf{y}} = \frac{1}{N}\sum_{j=1}^{N} \mathbf{y}_j. \tag{17}$$

For absolute error $e = |\hat{\mathbf{y}} - \mathbf{y}'|$ and threshold δ, the Huber loss is

$$\mathcal{L}_\delta(e) = \begin{cases} \frac{1}{2}e^2, & e < \delta, \\ \delta\big(e - \frac{1}{2}\delta\big), & e \geq \delta. \end{cases} \tag{18}$$

The weighted batch loss is

$$\mathcal{L}_{\text{Huber}} = \frac{1}{N}\sum_{i=1}^{N} w_i\,\mathcal{L}_\delta\big(|\hat{\mathbf{y}}_i - \mathbf{y}_i'|\big)\,. \tag{19}$$

Advanced Sampling and Resampling

Geographic Density-Based Sample Weighting

Weights w_i are inversely related to local sampling density. Examples include:

- **k-means cluster weights:** if $\mathbf{x}_i \in \mathcal{C}_k$ then $w_{i,\text{kmeans}} = \frac{1}{|\mathcal{C}_k|+\varepsilon}$.
- **KDE-based weights:** with density estimate $\hat{\rho}(\mathbf{x}_i)$, $w_{i,\text{kde}} = \big(\hat{\rho}(\mathbf{x}_i) + \varepsilon\big)^{-\alpha}$.

Final weights may combine the enabled methods and can be adjusted for focus regions.

Training Procedure Enhancements

Gradient Clipping

For gradient $\mathbf{g} = \nabla_\theta \mathcal{L}$ and threshold c:

$$\mathbf{g} \leftarrow \begin{cases} \mathbf{g}, & \|\mathbf{g}\|_2 \le c, \\ \dfrac{c}{\|\mathbf{g}\|_2}\,\mathbf{g}, & \|\mathbf{g}\|_2 > c. \end{cases} \tag{20}$$

Learning Rate Scheduling

A ReduceLROnPlateau scheduler reduces the learning rate η by factor f after p_{lr} epochs without improvement in validation loss.

Early Stopping

Stop training if for p_{es} consecutive epochs the validation loss does not improve by at least δ relative to the best value $\mathcal{L}_{\text{best}}$. The model state at $\mathcal{L}_{\text{best}}$ is retained.

References

Chawla, N. V., Bowyer, K. W., Hall, L. O., and Kegelmeyer, W. P. (2002). SMOTE: Synthetic minority over-sampling technique. *Journal of Artificial Intelligence Research*, 16, 321–357. `https://doi.org/10.1613/jair.953`.
